# Memory and comprehension of narrative versus expository texts: A meta-analysis

**DOI:** 10.3758/s13423-020-01853-1

**Published:** 2021-01-06

**Authors:** Raymond A. Mar, Jingyuan Li, Anh T. P. Nguyen, Cindy P. Ta

**Affiliations:** Department of Psychology, York University, 4700 Keele St. W., Toronto, ON M3J1P3 Canada

**Keywords:** Narrative texts, Expository texts, Story, Memory, Comprehension, Recall

## Abstract

We acquire a lot of information about the world through texts, which can be categorized at the broadest level into two primary genres: narratives and exposition. Stories and essays differ across a variety of dimensions, including structure and content, with numerous theories hypothesizing that stories are easier to understand and recall than essays. However, empirical work in this area has yielded mixed results. To synthesize research in this area, we conducted a meta-analysis of experiments in which memory and/or comprehension of narrative and expository texts was investigated. Based on over 75 unique samples and data from more than 33,000 participants, we found that stories were more easily understood and better recalled than essays. Moreover, this result was robust, not influenced by the inclusion of a single effect-size or single study, and not moderated by various study characteristics. This finding has implications for any domain in which acquiring and retaining information is important.

## Introduction

Reading is an important part of everyday life, as it is often the way in which we acquire new information (Stanovich & Cunningham, 1993). The texts we read take a variety of different forms, however, with the two broadest genres being narrative and expository texts. Stories and essays differ in many ways, including how they present and organize content. This has led many to theorize that narrative and expository texts might differ in their potential for readers to retain and comprehend the information presented. More specifically, a number of theories predict that narratives should be easier to recall and comprehend than expository texts. However, empirical examinations of this idea have been mixed. Some studies find this theorized advantage for stories in terms of memory and comprehension, but others have found an advantage for essays or no difference at all. We therefore conducted a meta-analysis to synthesize the available literature and uncover whether there is overall support for a difference in the memorability and comprehensibility of narrative and expository texts.

## Narrative versus expository texts

Narrative texts are written stories that most often take the form of novels or short stories. These have the goal of entertaining readers (Weaver & Kintsch, 1991) and possess a familiar structure. Events are focused on the actions, interactions, and development of characters, with these events organized based on temporal sequence and causal relations (Graesser, Golding, & Long, 1991; Tun, 1989; Zabrucky & Moore, 1999; Zabrucky & Ratner, 1992). Story events can thus be considered to follow a set structure known as a story grammar (Graesser et al., 1991; Kintsch, 1982), which includes the setting, theme, plot, and resolution (Thorndyke, 1977). Setting refers to the story’s time and place (Graesser et al., 1991; Graesser & Goodman, 1985), with the plot centred around the goals of the central character (i.e., the protagonist); these goals drive character actions and emotional reactions. In a story, the goals of different characters inevitably conflict, creating a tension that builds to a climax (De Beaugrande & Colby, 1979), followed by a resolution in which goals are either achieved or remain out of reach (Graesser et al., 1991). In this way, stories possess a clear and familiar structure, most commonly progressing through a chronological order of goal-centred events (Berman & Nir-Sagiv, 2007).

Expository texts, in contrast, are primarily intended to inform rather than entertain, communicating information and ideas about a specific topic (Decker, 1974; Graesser et al.,1991; Medina & Pilonieta, 2006). These texts can take the form of essays, textbooks, or manuals (Kintsch, 1982; Tun, 1989; Weaver & Kintsch, 1991), and contain descriptions, definitions, ideas, and explanations that are structured and supported by arguments (Boscolo, 1990; Mosenthal, 1985). The structure of exposition often resembles a pyramid, with the theme introduced first (i.e., the tip of the pyramid) and this theme subsequently elaborated on at length (Collins & Gentner, 1980; Graesser & Goodman, 1985).

## Theoretical differences between narratives and exposition

Based on these differences between stories and essays, researchers have long theorized that narratives might have an advantage over expository texts when it comes to memory and comprehension. Stories are more familiar than essays in many ways, including their resemblance to everyday experience, prevalence throughout human history, and precedence developmentally. In addition, stories are often more emotional than essays, and emotion can aid memory.

Stories may be easier to remember and comprehend than essays because stories resemble our everyday experiences (Bruner, 1986; Graesser et al., 1991). People experience life in the real world as temporally ordered causal events, organized around personal goals, with the encountering and overcoming of obstacles to these goals resulting in emotional experiences; this parallels the structure of stories (Graesser, McNamara, & Louwerse, 2003; Graesser, Singer, & Trabasso, 1994). In contrast, expository texts employ different structures depending on their purpose (Meyer, 1985), making them less familiar and less predictable. Exacerbating this problem, essays rarely contain the necessary linguistics markers that connect ideas and provide cues regarding the organization of content (e.g., connectives such as “because”; Graesser et al., 2003).

Not only the structure but also the content of stories map closely onto our everyday experiences. Stories are predominantly about social relationships: human psychology, interpersonal interactions, and the conflicts that inevitably result from conflicting goals (Mar & Oatley, 2008). As a result, the most common themes of stories are intimately familiar to us, topics such as friendship, interpersonal conflict, love, and separation from close others (Hogan, 2003; McNamara, Ozuru, & Floyd, 2017). Readers have direct, or indirect, experience with these topics and possess ample knowledge of these situations as a result (Gardner, 2004). This includes the vocabulary employed to describe these situations (e.g., words for traits, conflicts, and emotions) as they are all things we discuss in everyday life (Gardner, 2004). This close parallel between narratives and how we communicate our own experiences has led to stories being described as close to the “language of the mother tongue” (Graesser & Goodman, 1985).

In contrast, the content of exposition is often less familiar than what is found in stories, making it more difficult to comprehend and recall. Expository texts often communicate ideas that are new to the reader, and as a result they can contain unfamiliar concepts and vocabulary (Graesser et al., 2003; Weaver & Kintsch, 1991; Zabrucky & Moore, 1999). Furthermore, the content of essays is often complex and abstract, often focusing on situations that readers have not experienced (directly or indirectly) (Best, Floyd, & McNamara, 2008; Graesser et al., 2003; Hall, Sabey, & McClellan, 2005). Because essay content tends not to directly reflect everyday human experience, the vocabulary employed is often informational, scientific, and content-based, and therefore more difficult to understand than that found in stories (Gardner, 2004).

Familiarity with the structure and content of a text is referred to as relevant “prior knowledge” (Dochy, Segers, & Buehl, 1999), and stories might be more memorable and comprehensible thanks to readers having greater prior knowledge. Prior knowledge aids in the generation of inferences that support comprehension (Shapiro, 2004; Trabasso & Magliano, 1996) and recall. Readers generate more knowledge-based inferences when reading narratives compared to exposition (Clinton et al., 2020; Graesser & Clark, 1985), with these inferences explaining events in a text, bringing coherence to the content (Graesser et al., 1994; Trabasso & Magliano, 1996). For example, in a story, a reader can easily infer that a character will feel hurt if not invited to a party held by friends, without the author having to state this explicitly. This understanding stems from our familiarity with human psychology, even if only through second-hand experiences.

Readers are less likely to benefit from prior knowledge while reading an essay, relative to stories, and are therefore less likely to benefit from easy inferences. This combination could easily put expository texts at a disadvantage when it comes to memory and comprehension (Coté, Goldman, & Saul, 1998; McNamara, 2004). Readers often encounter expository texts when they do not know much about the content topic (Grabe, 2002). In schools, for example, readers are expected to learn new concepts from expository texts, based on little prior knowledge (Armbruster & Nagy, 1992; Barton, 1997; Grabe, 2002). With expository texts, it is rare that readers can rely on common knowledge to generate inferences. Rather, readers must rely on content knowledge of the domain in question (Graesser et al., 2003), potentially making essays harder to understand and recall than stories (Graesser et al., 2003; McKeown, Beck, Sinatra, & Loxterman, 1992; McNamara, Kintsch, Songer, & Kintsch, 1996).

Narratives are not only more familiar than essays as a function of their parallel with human experience, they also occupy a more prominent and familiar role throughout human history (Graesser et al., 1991; Graesser & Ottati, 1995). Before written texts existed, oral storytelling was the primary mode of communication, used to retain and transmit information from generation to generation (Graesser & Ottati, 1995; Rubin, 1995; Schank & Abelson, 1995). Stories were the basis of oral traditions and human memory was the sole vehicle for preserving these traditions, through frequent retelling (Graesser & Ottati, 1995; Rubin, 1995). For these reasons, stories and storytelling may have afforded our early ancestors with key benefits, including the dissemination of survival-relevant information (Bietti, Tilston, & Bangerter, 2019; Boyd, 2009; Scalise Sugiyama, 2001). Importantly, it is its resemblance to human experience that likely made stories so memorable, and so effective at disseminating complex surivival knowledge across generations of ancestors.

Stories also hold precedence over exposition at the timescale of individual development, perhaps resulting in greater familiarity. We are exposed to stories from the very beginning, from early childhood, often before we even have the capacity to speak or read (Baker & Stein, 1978; Spiro & Taylor, 1987). This early exposure to narratives continues throughout childhood, with narratives being the most common type of text encountered during early schooling (Leslie & Caldwell, 2017). In contrast, there is a relative lack of early exposure to expository texts, with students first being exposed to essays around third grade and onwards (around ages 8–9 years; Spiro & Taylor, 1987). From this point, students increasingly encounter expository texts as they progress through school and, eventually, exposition becomes the predominant type of text in high school (Kent, 1984). Their late introduction might be another reason why expository texts could be less familiar, and therefore less likely to be remembered and comprehended compared to narrative texts.

A final reason to believe that narratives may be more memorable than expository texts hinges on the ability of emotions to facilitate memory (Hamann, 2001). Affectively charged recollections have been dubbed “flash-bulb” memories, to communicate the idea that emotional events are deeply imprinted on the mind, like a flash aiding photography (Winograd & Neisser, 1992). This emotional facilitation of memory appears to result from a prioritizing of emotional material when it comes to attention and perception (Brosch, Pourtois, & Sander, 2010), with personal relevance playing a key role (Levine & Edelstein, 2009). To the extent that stories are better able to evoke strong emotions than expository texts (cf. Mar, Oatley, Djikic, & Mullin, 2011), we would expect stories to be better recalled than exposition. The idea that stories are emotional in nature seems obvious, so much so that this is simply assumed by lay people and researchers alike (Oatley, 1991). Researchers, for example, use stories to elicit mood for experimental manipulations (e.g., Kazui et al., 2000). Empirical evidence for the emotional nature of stories also exists, with one diary study finding that roughly 7% of all emotions were elicited while engaging with narrative (Oatley & Duncan, 1992). Similarly, readers experience and mentally represent the emotional states of story protagonists (Gernsbacher, Goldsmith, & Robertson, 1992; Laszlo & Cupchik, 1995; Oatley, 1999), and report emotions occurring frequently while reading (Larsen & Seilman, 1988). Notably, studies on emotional memory have also employed stories as stimuli and confirmed that emotional content is better remembered than neutral content (e.g., Cahill, Babinsky, Markowitsch, & McGaugh, 1995; Carstensen & Turk-Charles, 1994; Kazui et al., 2000; McGaugh, 2000). It is difficult to imagine that expository texts, in general, have the capacity to elicit emotions to the same extent, or with the same variety, as narratives. This is because exposition lacks the close parallel with human experiences found in stories. Lastly, it should be stressed that this emotional account of why stories might be better understood and remembered than essays is not mutually exclusive to the structural and organizational accounts presented above. Several factors could play independent roles in any observed advantage for narrative. In addition, it should also be noted that not all texts fit easily into these broad categories, such as narrative journalism, which bridges the two approaches (van Krieken & Sanders, in press).

## Empirical research on narrative and expository texts

In light of these theoretical advantages for narrative over exposition, when it comes to memory and comprehension, a number of researchers have investigated this topic using experiments. To do so, researchers randomly assign participants to read either a narrative or an expository passage (a between-subjects design), or read both (a within-subjects design). In some studies, participants listen to audio versions of these texts, rather than read them. But in all cases, comprehension and memory for the texts is measured. Unfortunately, these experiments have yielded mixed results. Some studies do indeed find greater recall or comprehension of narrative texts relative to expository texts (e.g., Best et al., 2008; Dal Martello, 1984; Tun, 1989; Zabrucky & Moore, 1999). In contrast, other studies find just the opposite: that expository texts are more easily comprehended and better recalled than narratives (e.g., Diakidoy, 2014; Moè & De Beni, 2005; Saadatnia, Ketabi, & Tavakoli, 2017; Wolfe & Woodwyk, 2010). A few studies also report finding no difference between the two genres (e.g., Cunningham & Gall, 1990; Kintsch & Young, 1984; Roller & Schreiner, 19856). Based on these conflicting results, it is evident that a meta-analysis is necessary to establish whether it is possible to detect an overall effect based on the extant evidence. A recent meta-analysis on inferential comprehension found that narrative had an advantage over exposition (Clinton et al., 2020). Here, we report the results of a broader, more inclusive meta-analysis, synthesizing the results of existing studies for both memory and comprehension, to uncover whether narrative and exposition differ in this regard.

## Method

### Identifying and retrieving articles

To identify relevant empirical papers, an extensive literature search was conducted in August 2018. This was then updated in November 2019, when unpublished articles were also solicited from listservs. We systematically searched the following online databases for suitable articles: PsycINFO, PsycARTICLES, and Web of Science. In each database, we searched the following terms: narrat* OR *story* AND exposit* OR *prose* OR *essay* OR *summary* AND *recall* OR *retention* OR *recognition* OR *remember* OR *comprehension* OR *comprehend* OR *schema* OR *retrieval.* Results were limited to articles published in English. When possible, the search was confined to empirical studies (i.e., PsycINFO and PsychARTICLES) or journal articles (for Web of Science).

The first search in 2018 yielded a total of 871 articles, with the removal of duplicates resulting in 689 unique papers, including two articles added based on our own expert knowledge. This search was repeated in November 2019 to locate any new papers published since the previous search. This second search employed the same search terms used previously, but was limited to the time period following the previous one. However, it did not result in the identification of any new articles. At this time, we also solicited unpublished work on this topic from several academic listservs (i.e., the *Society for Text and Discourse*, the *Psychonomic Society*, the *International Society for the Empirical Study of Literature and Media*, and the *UK Literary Association*). We were consequently able to include two unpublished studies, thanks to the generous collaboration of other researchers.

### Inclusion and exclusion criteria

To be eligible for our meta-analysis, a study was required to meet a set of inclusion criteria. Included studies had to: (1) allow for the comparison of memory and/or comprehension performance between narrative and expository texts; (2) be a true experiment, with proper random assignment and counterbalancing; (3) include a measure of memory (e.g., immediate, delayed, free, or cued recall) or comprehension (e.g., open-ended or closed comprehension questions, sentence verification); and (4) examine non-clinical populations (i.e., no special populations, such as those with a reading disorder).

A set of exclusion criteria was also established. Studies were excluded if: (1) text order was not randomized for a within-subjects design (or randomization was not explicitly mentioned), producing a confound between genre and order; (2) the two genres were read at very different points in time for a within-subjects experiment (e.g., on different days), creating a potential confound between genre and time (i.e., history effects; Campbell & Stanley, 1963); (3) studies employed different measures of memory or comprehension for the two genres (e.g., free recall tested for stories, but cued recall for essays); (4) the procedures did not approximate typical leisure reading (e.g., asking participants to read aloud or focus on certain story elements); and (5) no relevant statistics for our purposes were reported (i.e., it was impossible to calculate the requisite effect-size). Although our primary interest was reading, we also included studies employing auditory presentations so that we could explore whether presentation modality moderates any effects.

### Coding procedure

Articles were screened for inclusion and coded in early 2019, extracting the statistics required to calculate an effect-size. This entailed the means and standard deviations of task scores, recorded as percentage correct to allow for a direct comparison between text genres. When studies reported standard errors instead of standard deviations, the former were converted into the latter using the following formula: $\mathrm{SD}=\mathrm{SE}\times \sqrt{\mathrm{n}}.$

To maximize the information gleaned from each study, all possible relevant comparisons were extracted. For example, if a study employed two measures of memory, genre comparisons for both measures were extracted. Similarly, if the study reported separate statistics for subsets of the sample (e.g., male scores and female scores), these sub-scores were chosen instead of the aggregate. When experiments employed a control condition with no intervention, both pre-test and post-test scores were taken. However, if an experimental intervention was included (e.g., to improve comprehension), only pre-test scores were taken. As a result of this inclusive approach, each article yielded several relevant comparisons. Our statistical approach to meta-analysis models the dependence between effect-sizes, making it possible to include multiple effects per sample.

In addition, we coded several aspects of each study to examine potential moderators. This included information about the study design (e.g., between- or within-subjects), demographic variables (e.g., age of participants), stimuli characteristics (e.g., whether researchers attempted to control for content or difficulty), and dependent variables (e.g., delay between reading and testing). Table 1 provides a description of all the moderator variables that were included in the final analysis.

**Table 1:** Potential moderators

Moderator	Description
Memory or comprehension	Whether the effect-size pertains to the memory or comprehension of the narrative and expository texts, as reported by the authors
Immediate or delay	Whether the test of memory or comprehension occurred immediately or after a delay
Adults or non-adults	Whether the sample consisted of adults (17+ years) or non-adults (16 years and younger)
Read or heard	Whether the participants read (e.g., on paper, a computer screen) or heard the texts (e.g., spoken by an individual or computer)
Content controlled	Whether there was an attempt to control the content across the narrative and expository texts, as reported by the authors
Difficulty controlled	Whether there was an attempt to control the reading difficulty across the narrative and expository texts (e.g., Flesch-Kincaid Grade level, Fry Readability Level), as reported by the authors
Verbal or written test	Whether the memory or comprehension test presented was verbal (e.g., tape-recording or note-taking of free recall, then coded for accuracy) or written (e.g., multiple-choice questions)

### Coding outcome

**Fig. 1:**
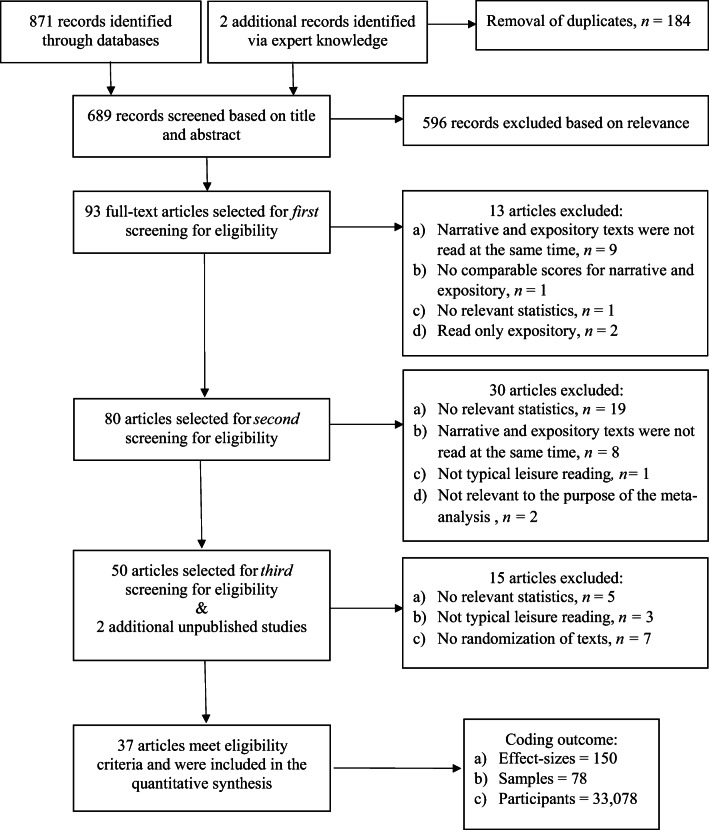
Process for identifying and selecting studies

Titles and abstracts for the 689 papers were first examined for relevance, resulting in 93 articles selected for closer reading. Based on full-text screening, 80 articles were considered for inclusion in the meta-analysis. Screening and coding of these 80 articles was carried out by 13 independent coders, who each coded a subset after receiving extensive training. Any uncertainty during coding was discussed among the group and a consensus was established. Following the second literature search in 2019, all coding was double-checked and a consensual coding again established. In the end, statistics from a total of 37 articles were extracted, resulting in the compilation of 150 separate effect-sizes, based on 78 different samples, for a total sample of 33,078 participants (Table 2). Figure 1 illustrates our process. All of our data are publicly available at: https://osf.io/jx78v/.

**Table 2:** Descriptive statistics for effect-sizes

ID	Author(s), year	Outcome	Sample size N	Narrative *M (SD)*	Expository *M* (*SD*)
1	Panico & Healey, 2009	Memory	15	0.55 (0.08)	0.47 (0.10)
2	Panico & Healey, 2009	Memory	15	0.86 (0.11)	0.64 (0.13)
3	Panico & Healey, 2009	Comprehension	15	0.88 (0.11)	0.78 (0.13)
4	Carnine & Kinder, 1985	Comprehension	14	0.49 (0.21)	0.41 (0.23)
5	Carnine & Kinder, 1985	Comprehension	13	0.53 (0.18)	0.36 (0.24)
6	Luszcz, 1993a	Memory	120	0.34 (0.12)	0.18 (0.10)
7	Tun, 1989	Memory	20	0.51 (0.11)	0.41 (0.13)
8	Tun, 1989	Memory	20	0.62 (0.11)	0.44 (0.14)
9	Tun, 1989	Memory	20	0.45 (0.12)	0.38 (0.14)
10	Tun, 1989	Comprehension	20	0.92 (0.05)	0.88 (0.80)
11	Tun, 1989	Memory	20	0.31 (0.09)	0.19 (0.09)
12	Tun, 1989	Memory	20	0.41 (0.11)	0.23 (0.11)
13	Tun, 1989	Memory	20	0.26 (0.09)	0.16 (0.10)
14	Tun, 1989	Comprehension	20	0.93 (0.07)	0.75 (0.09)
15	Waddill, McDaniel, & Einstein, 1988	Memory	24	0.42 (0.23)	0.12 (0.14)
16	Waddill et al., 1988	Memory	24	0.38 (0.15)	0.21 (0.16)
17	Waddill et al., 1988	Memory	24	0.34 (0.09)	0.13 (0.15)
18	Waddill et al., 1988	Memory	24	0.62 (0.26)	0.42 (0.17)
19	Waddill et al., 1988	Memory	24	0.26 (0.16)	0.21 (0.15)
20	Best et al., 2008	Memory	61	0.10 (0.07)	0.04 (0.03)
21	Best et al., 2008	Memory	61	0.15 (0.10)	0.07 (0.03)
22	Best et al., 2008	Comprehension	61	0.72 (0.17)	0.49 (0.15)
23	Weaver & Bryant, 1995	Comprehension	98	0.65 (0.14)	0.45 (0.21)
24	Weaver & Bryant, 1995	Comprehension	98	0.64 (0.21)	0.39 (0.21)
25	Sadoski, Goetz, & Rodriguez, 2000	Memory	80	0.24 (0.16)	0.20 (0.17)
26	Sadoski et al., 2000	Memory	80	0.34 (0.14)	0.23 (0.17)
27	Sadoski et al., 2000	Memory	80	0.29 (0.19)	0.25 (0.16)
28	Sadoski et al., 2000	Memory	80	0.37 (0.17)	0.33 (0.19)
29	Sadoski et al., 2000	Memory	80	0.24 (0.16)	0.15 (0.12)
30	Sadoski et al., 2000	Memory	80	0.34 (0.14)	0.19 (0.13)
31	Sadoski et al., 2000	Memory	80	0.29 (0.19)	0.33 (0.15)
32	Sadoski et al., 2000	Memory	80	0.37 (0.17)	0.33 (0.13)
33	Margolin, Driscoll, Toland, & Kegler, 2013	Comprehension	30	0.74 (0.12)	0.80 (0.12)
34	Margolin et al., 2013	Comprehension	30	0.76 (0.14)	0.79 (0.13)
35	Margolin et al., 2013	Comprehension	30	0.73 (0.15)	0.75 (0.14)
36	Wolfe, 2005	Memory	144	0.38 (0.10)	0.24 (0.06)
37	Diakidoy, Stylianou, Karefillidou, & Papageorgiou, 2005	Comprehension	125	0.60 (0.19)	0.59 (0.16)
38	Diakidoy et al., 2005	Comprehension	132	0.75 (0.18)	0.64 (0.16)
39	Diakidoy et al., 2005	Comprehension	142	0.80 (0.17)	0.68 (0.17)
40	Diakidoy et al., 2005	Comprehension	163	0.78 (0.16)	0.73 (0.16)
41	Diakidoy et al., 2005	Comprehension	125	0.69 (0.17)	0.61 (0.17)
42	Diakidoy et al., 2005	Comprehension	132	0.74 (0.18)	0.62 (0.15)
43	Diakidoy et al., 2005	Comprehension	142	0.82 (0.17)	0.67 (0.18)
44	Diakidoy et al., 2005	Comprehension	163	0.73 (0.17)	0.70 (0.15)
45	Diakidoy, 2014	Comprehension	90	0.60 (0.21)	0.67 (0.18)
46	Diakidoy, 2014	Comprehension	90	0.66 (0.18)	0.68 (0.17)
47	Olson, 1985	Comprehension	27	0.35 (0.08)	0.22 (0.12)
48	Olson, 1985	Comprehension	27	0.36 (0.05)	0.24 (0.08)
49	Olson, 1985	Comprehension	27	0.34 (0.07)	0.23 (0.09)
50	Olson, 1985	Comprehension	26	0.29 (0.10)	0.15 (0.15)
51	Olson, 1985	Comprehension	26	0.30 (0.09)	0.16 (0.07)
52	Olson, 1985	Comprehension	26	0.26 (2.05)	0.16 (0.09)
53	Wolfe & Mienko, 2007	Memory	90	0.34 (0.11)	0.31 (0.11)
54	Wolfe & Mienko, 2007	Memory	90	0.34 (0.11)	0.30 (0.10)
55	Wolfe & Mienko, 2007	Comprehension	90	0.17 (0.16)	0.20 (0.17)
56	Wolfe & Mienko, 2007	Comprehension	90	0.17 (0.16)	0.18 (0.13)
57	Dickens & Meisinger, 2017	Comprehension	44	0.55 (0.23)	0.44 (0.25)
58	Dickens & Meisinger, 2017	Comprehension	43	0.43 (0.22)	0.39 (0.22)
59	Dickens & Meisinger, 2017	Comprehension	43	0.45 (0.22)	0.32 (0.18)
60	Dickens & Meisinger, 2017	Comprehension	43	0.39 (0.23)	0.29 (0.19)
61	Harris, Rogers, & Qualls, 1998	Comprehension	27	0.95 (0.09)	0.93 (0.12)
62	Harris et al., 1998	Comprehension	27	0.97 (0.07)	0.94 (0.09)
63	Harris et al., 1998	Comprehension	27	0.81 (0.17)	0.93 (0.11)
64	Harris et al., 1998	Comprehension	27	0.76 (0.18)	0.93 (0.17)
65	Harris et al., 1998	Comprehension	27	0.94 (0.08)	0.94 (0.09)
66	Harris et al., 1998	Comprehension	27	0.96 (0.08)	0.93 (0.11)
67	Harris et al., 1998	Comprehension	27	0.90 (0.16)	0.96 (0.07)
68	Harris et al., 1998	Comprehension	27	0.80 (0.16)	0.94 (0.14)
69	Harris et al., 1998	Comprehension	27	0.94 (0.09)	0.91 (0.12)
70	Harris et al., 1998	Comprehension	27	0.98 (0.06)	0.92 (0.09)
71	Harris et al., 1998	Comprehension	27	0.86 (0.16)	0.96 (0.12)
72	Harris et al., 1998	Comprehension	27	0.74 (0.20)	0.91 (0.11)
73	Harris et al., 1998	Comprehension	27	0.94 (0.10)	0.95 (0.08)
74	Harris et al., 1998	Comprehension	27	0.98 (0.06)	0.93 (0.14)
75	Harris et al., 1998	Comprehension	27	0.90 (0.13)	0.93 (0.13)
76	Harris et al., 1998	Comprehension	27	0.77 (0.22)	0.94 (0.10)
77	Lehto & Anttila, 2003	Comprehension	22	0.82 (0.11)	0.78 (0.10)
78	Lehto & Anttila, 2003	Comprehension	15	0.79 (0.10)	0.72 (0.10)
79	Lehto & Anttila, 2003	Comprehension	18	0.88 (0.09)	0.79 (0.09)
80	Lehto & Anttila, 2003	Comprehension	13	0.86 (0.09)	0.79 (0.09)
81	Lehto & Anttila, 2003	Comprehension	21	0.89 (0.12)	0.83 (0.12)
82	Lehto & Anttila, 2003	Comprehension	18	0.88 (0.10)	0.76 (0.12)
83	Mulholland & Neville, 1989	Memory	158	0.49 (0.21)	0.36 (0.25)
84	Mulholland & Neville, 1989	Memory	228	0.39 (0.22)	0.17 (0.25)
85	Mulholland & Neville, 1989	Memory	223	0.68 (0.16)	0.63 (0.21)
86	Mulholland & Neville, 1989	Memory	213	0.61 (0.16)	0.40 (0.19)
87	Mulholland & Neville, 1989	Memory	211	0.68 (0.17)	0.66 (0.18)
88	Mulholland & Neville, 1989	Memory	227	0.68 (0.14)	0.49 (0.21)
89	Mulholland & Neville, 1989	Memory	207	0.46 (0.20)	0.38 (0.22)
90	Mulholland & Neville, 1989	Memory	197	0.39 (0.18)	0.23 (0.13)
91	Mulholland & Neville, 1989	Memory	211	0.65 (0.18)	0.62 (0.20)
92	Mulholland & Neville, 1989	Memory	205	0.61 (0.15)	0.33 (0.15)
93	Mulholland & Neville, 1989	Memory	219	0.71 (0.17)	0.67 (0.18)
94	Mulholland & Neville, 1989	Memory	209	0.63 (0.17)	0.38 (0.16)
95	Padeliadu & Antoniou, 2014	Comprehension	114	0.20 (0.13)	0.05 (0.07)
96	Padeliadu & Antoniou, 2014	Comprehension	113	0.35 (0.16)	0.13 (0.10)
97	Padeliadu & Antoniou, 2014	Comprehension	134	0.65 (0.06)	0.40 (0.09)
98	Padeliadu & Antoniou, 2014	Comprehension	108	0.66 (0.04)	0.42 (0.07)
99	Padeliadu & Antoniou, 2014	Comprehension	108	0.68 (0.04)	0.46 (0.07)
100	Valencia & Stallman, 1989	Comprehension	200^a^	0.80 (0.19)	0.64 (0.21)
101	Valencia & Stallman, 1989	Comprehension	200^a^	0.79 (0.16)	0.56 (0.30)
102	Valencia & Stallman, 1989	Comprehension	200^a^	0.65 (0.19)	0.59 (0.17)
103	Valencia & Stallman, 1989	Comprehension	200^a^	0.71 (0.19)	0.72 (0.22)
104	Pomplun & Omar, 2001	Comprehension	12562	0.84 (0.10)	0.75 (0.11)
105	Pomplun & Omar, 2001	Comprehension	12102	0.79 (0.12)	0.73 (0.12)
106	Dai & Wang, 2007	Comprehension	233	0.62 (0.21)	0.53 (0.24)
107	Dai & Wang, 2007	Comprehension	233	0.62 (0.21)	0.57 (0.27)
108	Primor, Pierce, & Katzir, 2011	Comprehension	190	-2.18 (0.60)^b^	-2.48 (0.53)^b^
109	Wightman & Roney, 2013	Comprehension	10	0.66 (0.17)	0.69 (0.12)
110	Wightman & Roney, 2013	Comprehension	11	0.68 (0.17)	0.76 (0.17)
111	Wightman & Roney, 2013	Comprehension	10	0.76 (0.16)	0.75 (0.16)
112	Wightman & Roney, 2013	Comprehension	13	0.79 (0.17)	0.76 (0.17)
113	Margolin & Hover, 2011	Comprehension	62	0.71 (0.20)	0.73 (0.21)
114	Margolin & Hover, 2011	Comprehension	62	0.66 (0.24)	0.49 (0.27)
115	Margolin & Hover, 2011	Comprehension	62	0.74 (0.22)	0.57 (0.23)
116	Margolin, Snyder, & Thamboo, 2018	Comprehension	60	0.67 (0.20)	0.56 (0.18)
117	Margolin et al., 2018	Comprehension	60	0.78 (0.16)	0.45 (0.23)
118	Margolin et al., 2018	Comprehension	60	0.75 (0.18)	0.51 (0.22)
119	Margolin et al., 2018	Comprehension	60	0.65 (0.21)	0.38 (0.26)
120	Simmons et al., 2014	Comprehension	432	0.81 (0.17)	0.61 (0.22)
121	Simmons et al., 2014	Comprehension	489	0.80 (0.17)	0.59 (0.23)
122	Hay & Moran, 2005	Memory	9	0.91 (0.10)	0.74 (0.12)
123	Hay & Moran, 2005	Memory	9	1.27 (0.20)^c^	0.88 (0.09)
124	Hay & Moran, 2005	Memory	9	0.56 (0.31)	0.39 (0.17)
125	Hay & Moran, 2005	Memory	9	0.62 (0.20)	0.54 (0.17)
126	Hay & Moran, 2005	Memory	9	0.69 (0.14)	0.62 (0.23)
127	Hay & Moran, 2005	Memory	9	0.78 (0.16)	0.64 (0.26)
128	Hay & Moran, 2005	Comprehension	9	0.78 (0.33)	0.85 (0.29)
129	De Beni, Palladino, Borella, & Presti, 2003	Comprehension	129	0.64 (0.16)	0.76 (0.18)
130	De Beni et al., 2003	Comprehension	121	0.55 (0.16)	0.65 (0.22)
131	Luszcz, Luszcz, 1993b	Memory	20	0.32 (0.07)	0.26 (0.13)
132	Luszcz, 1993b	Memory	20	0.34 (0.11)	0.13 (0.07)
133	Schroeder, 2011	Comprehension	119	0.53 (0.22)	0.51 (0.22)
134	Moè & De Beni, 2005	Memory	15	0.26 (0.10)	0.34 (0.07)
135	Moè & De Beni, 2005	Memory	15	0.18 (0.09)	0.19 (0.05)
136	Moè & De Beni, 2005	Memory	15	0.25 (0.09)	0.29 (0.08)
137	Moè & De Beni, 2005	Memory	15	0.24 (0.06)	0.21 (0.06)
138	Moè & De Beni, 2005	Memory	15	0.15 (0.04)	0.19 (0.07)
139	Moè & De Beni, 2005	Memory	15	0.23 (0.09)	0.31 (0.07)
140	Narvaez, Van Den Broek, & Ruiz, 1999	Comprehension	20	0.70 (0.24)	0.72 (0.22)
141	Guan, Ye, Wagner, Meng, & Leong, 2014	Comprehension	246	0.40 (0.19)	0.40 (0.16)
142	Guan et al., 2014	Comprehension	242	0.53 (0.21)	0.53 (0.20)
143	Guan et al., 2014	Comprehension	261	0.59 (0.21)	0.61 (0.19)
144	Cunningham & Gall, 1990	Comprehension	313	0.16 (0.12)	0.15 (0.11)
145	Hinze, 2015	Memory	46	0.89 (0.09)	0.95 (0.06)
146	Hinze, 2015	Comprehension	46	0.84 (0.11)	0.89 (0.09)
147	Hinze, 2015	Memory	38	0.75 (0.16)	0.78 (0.16)
148	Hinze, 2015	Comprehension	38	0.54 (0.18)	0.62 (0.20)
149	Rudiger & Hinze, 2017	Memory	36	0.18 (0.21)	0.20 (0.20)
150	Rudiger & Hinze, 2017	Memory	81	0.46 (0.23)	0.53 (0.24)

^a^ The sample is described as “approximately 600” with one third in the control condition; we used 200 for our calculations

^b^ Scores were transformed using a reflection and square-root procedure so that higher values indicate lower scores; we changed these to negative values (resulting in higher values indicating better scores)

^c^ Participants produced more T-units in their recall than included in the original texts

### Statistical analysis

We first calculated effect-sizes for all comparisons (Hedge’s *g*, with positive values indicating an advantage for narrative), then conducted a three-level random-effects meta-analysis of these effects. All analyses were done in R (version 3.5.1; R Core Team, 2018), based on a script provided by Dodell-Feder and Tamir (2018), with the help of the *metafor* package (Viechtbauer, 2010).

#### Three-level meta-analysis

Because most articles contained multiple comparisons (and therefore multiple effect-sizes), in addition to multiple studies per article in some cases, it is necessary to model the nested nature of these data. It is likely that effect-sizes drawn from the same study are intercorrelated, and this dependency must be taken into account. To incorporate multiple dependent effect-sizes, we used a three-level random-effects meta-analysis model, accounting for variance among the effect-sizes (level 1), variance in effect-sizes within a single study (level 2), and the variance between different studies (level 3). This three-level model mirrors the hierarchical structure of our data, clustering effect-sizes nested within a study. Importantly, the sampling error within clusters is dependent, due to the overlap in samples (e.g., comparisons between genres for both recall and comprehension, within a single study). To account for this dependency we calculated cluster-robust standard errors, statistical tests, and confidence intervals (CIs) for our estimates from the three-level model.

A high degree of variability among effect-sizes can tell us whether study characteristics influence the effects observed. This heterogeneity among effect-sizes can be quantified and assessed using the *Q* statistic. A statistically significant *Q* value tells us that effect-sizes differ from each other more than what is expected based on sampling error alone. As a result, we can conclude that differences in effect-size may be due to some aspect of the studies. The main shortcoming of the *Q* statistic is that it does evaluate the extent of heterogeneity observed, assessing only its presence or absence (Huedo-Medina, Sánchez-Meca, Marín-Martínez, & Botella, 2006). To address this shortcoming, we use τ^2^ to quantify the heterogeneity for level 2 (within studies) and level 3 (between studies) of our meta-analysis (Cheung, 2014). Large τ^2^ values indicate that a large amount of variance in effect-sizes is not due to chance and might be caused by other factors that should be investigated, using a moderator analysis for example. On the other hand, small τ^2^ values indicate that the effect-sizes are similar to one another, with differences between them likely due to chance. We estimated τ^2^ by using restricted maximum-likelihood estimation (REML), the default in the *metafor* package (Viechtbauer, 2010). Note that τ^2^ depends on the effect-size used, so unstandardized τ^2^ values are not comparable across meta-analyses (Huedo-Medina et al., 2006). Fortunately, Higgins and Thompson (2002) proposed the *I*^2^ index to overcome these shortcomings. The *I*^2^ index can be interpreted as the percentage of total variability that is due to true heterogeneity rather than sampling error. We report total *I*^2^, with *I*^2^_Level 2_ and *I*^2^_Level 3_ representing within- and between-study heterogeneity, respectively. Large *I*^2^ values indicate that a large proportion of the variance in effect-sizes is likely caused by systematic differences in study-level factors. This indicates that a moderator analysis may help to explain study-level variability. Low *I*^2^ values indicate that the variability in effect-sizes is small and likely due to chance. We interpret the *I*^2^ values by using the benchmarks provided by Higgins and Thompson (2002).

#### Sensitivity analyses

To examine whether our results are robust, and do not change based on small differences in what effect-sizes or studies are included, we conducted a series of sensitivity analyses. Effect-sizes that deviate markedly from others are potential outliers, with those that meaningfully impact coefficients known as *influential outliers*. These cases can distort results and lead to false conclusions. We defined influential outliers as effect-sizes with a standardized residual exceeding 3.0 that also have values for Cook’s distance exceeding .027, the latter based on the formula 4/(*n*-*k*-1), where *k* = number of predictors (Fox, 1991; as cited in Dodell-Feder & Tamir, 2018). If influential outliers exist, we planned to re-estimate our model after excluding them. To further examine the impact of each effect-size and study, we also conducted another sensitivity analysis: the leave-one-out procedure. This involves re-running the model multiple times, leaving out a different effect-size each time. A similar analysis was conducted leaving out one study each time. In this fashion, effect-sizes or studies whose inclusion dramatically influences the results can be identified.

#### Moderator analysis

If we find substantial variance among effect-sizes based on the *Q* statistic and *I*^2^ values, we can then ask whether this variability can be explained by systematic differences between studies. Observed heterogeneity in effect-sizes was formally investigated via moderator analyses, incorporating study characteristics that vary both within studies (e.g., outcome measures) and between studies (e.g., adults or non-adult participants). One study characteristic–type of outcome measure – can vary both within and between studies. To be more specific, six studies measured both recall and comprehension, whereas other studies only measured one of these outcomes. To investigate any possible confound introduced by differences between studies, we also conducted follow-up analyses using only those studies that measured both recall and comprehension to re-estimate the model.

#### Publication bias

Lastly, we examined the possibility of publication bias: that studies identifying a difference or effect are more likely to be published, skewing our results. To diagnose publication bias, we produced a funnel plot depicting the relation between effect-sizes and their standard errors, with the latter representing the precision of the effect-size estimates. More precise results should be at the top of the plot and cluster tightly around the true effect (i.e., the vertical line on the plot), whereas less precise studies should be at the bottom and scatter widely around the mean, forming a funnel shape. A lack of symmetry in the plot indicates that publication bias may exist (e.g., few studies on the left side), possibly inflating the estimated overall effect-size. Studies with statistically significant findings should be located on the right side, with an over-representation of studies at the right bottom indicating evidence for publication bias. This pattern represents the presence of statistically significant, but low-powered, findings with equally likely statistically nonsignificant findings not being published. Although funnel plots are informative, they do not account for the multilevel structure of our data, which can also lead to clustered portions on the plot and therefore produce asymmetry that could be misinterpreted as bias. For this reason, we also conducted an Egger's regression test by including the standardized error of the effect-sizes as a moderator in the three-level models. In other words, we evaluated whether the precision of the effect was related to the effect-size magnitude. If standardized error coefficients predict effect-size, this indicates that there is a systematic difference between effect-sizes from studies with low versus high precision, indicating the presence of publication bias. The code to replicate our analyses and reproduce our figures can be accessed here: https://osf.io/jx78v/.

## Results

### Meta-analysis

**Fig. 2:**
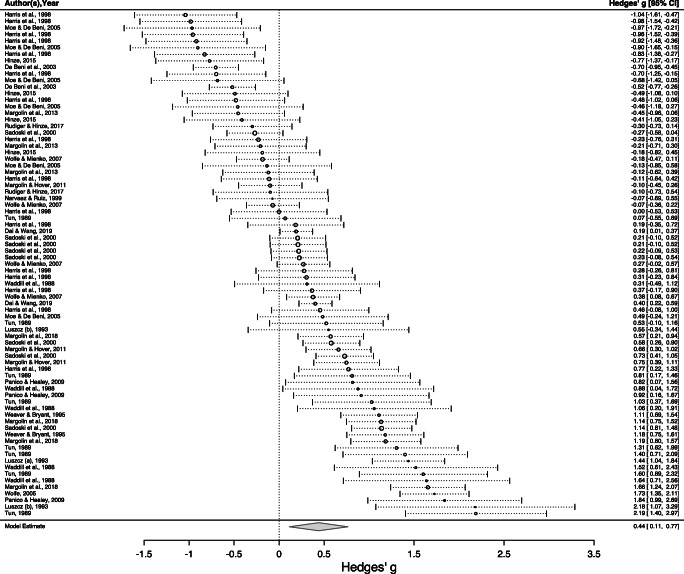
Forest plot for adult participants

**Fig. 3:**
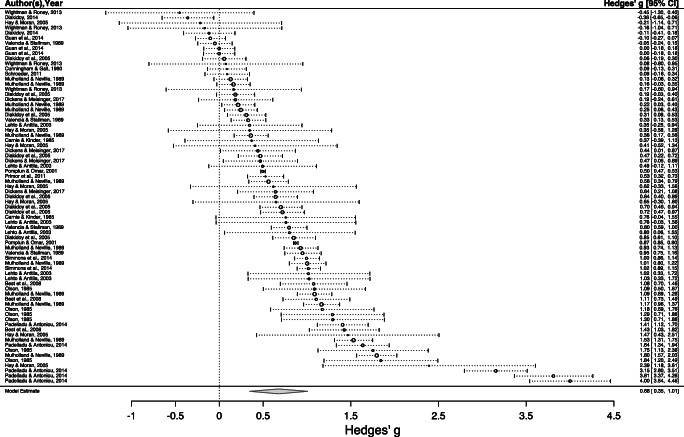
Forest plot for nonadult participants

Our primary research question was whether memory and comprehension differ for narrative versus expository texts. Our three-level random-effects meta-analysis of 150 effect-sizes found that, on average, memory and comprehension of narrative texts was superior to that for expository texts. The mean effect-size was a Hedge’s *g* of .55, with a 95% CI ranging from .31 to .79, *p* < .001 (Table 3). Thus, the average size of this effect was estimated to be just more than half a standard deviation in magnitude. Forest plots summarizing all effects are presented for our two main sample groups, adults (17 or more years of age) and non-adults (≦ 16 years) (Figs. 2 and 3).

**Table 3:** Meta-analysis results

Variable	Number of studies	Effect-sizes	*g*	95% CI	*SE*	*t*	*p*	*Q*
Overall estimate	37	150	.55^***^	.31, .79	.12	4.66	< .001	2884.68^***^
Dependent variable						1.73	.09	2884.64^***^
Comprehension	29	93	.48^**^	.21, .75	.13			
Memory	14	57	.72^***^	.43, 1.01	.14			
Participants						1.00	.32	2642.38^***^
Adults	19	77	.44^*^	.10, .77	.17			
Non-adults	18	73	.67^***^	.33, 1.01	.17			
Modality						.66	.52	2884.52^***^
Listen	8	32	.61^***^	.32, .90	.14			
Read	34	118	.54^**^	.30, .79	.12			
Content						.05	.96	2881.81^***^
Not controlled	29	108	.55^***^	.31, .79	.12			
Controlled	8	42	.57	-.13, 1.27	.34			
Difficulty						.13	.90	2866.57^***^
Not controlled	21	95	.54^***^	.27, .81	.13			
Controlled	15	55	.57^*^	.13, 1.02	.22			
Immediate/delayed						.07	.95	2845.80^***^
Delayed	4	14	.53	-.31, 1.36	.41			
Immediate	32	136	.56^***^	.30, .81	.13			
Test format						1.98	.06	2835.17^***^
Verbal	6	21	.92^***^	.59, 1.26	.17			
Written	31	129	.49^***^	.22, .76	.13			

Number of studies within a moderator variable may exceed *N* = 37 as some studies contained both levels of the moderator

^*^p < .05, ^**^p < .01, ^***^p < .001

With respect to variability in these effect-sizes, the *Q* statistic was statistically significant, indicating the presence of heterogeneity, *Q*(149) = 2884.68, *p* < .001. The Total *I*^*2*^ was 98%, indicating a large proportion of true heterogeneity rather than sampling error, the majority of which came from between-study variance (*I*^*2*^_level 3_ = 67%), with within-study variance being relatively low, *I*^*2*^_level 2_ = 31%. Because these differences among effect-sizes are largely caused by factors that vary between studies, we examined possible moderators, after first establishing the robustness of our main finding with a series of sensitivity analyses.

### Sensitivity analyses

To ensure that our results are reliable and do not change as a function of small changes in what effect-sizes or studies are included, we conducted a series of sensitivity analyses. First, we examined whether there were any influential cases among our effect-sizes, but this process did not identify any influential outliers (based on the criteria described in our methods). Next, we performed a leave-one-out analysis at the level of individual effect-sizes. The meta-analysis was re-run multiple times, each time removing one effect-size, but the estimate of the overall effect barely changed (*g*_range_ = .54–.57). This analysis also found that the true variance of effect sizes remained substantial, lowest *I*^*2*^ = 97% (*I*^*2*^_Level 2_ = 35%, *I*^*2*^_Level 3_ = 63%); highest *I*^*2*^ = 98% (*I*^*2*^_Level 2_ = 30%, *I*^*2*^_Level 3_ = 68%). Likewise, the leave-one-out analysis at the study-level also illustrated that the effect was robust and not driven by any one particular study (*g*_range_ = .48–.59). The overall effect remained medium in size and statistically significant. In addition, the true variance of effect-sizes also remained substantial, lowest *I*^*2*^ = 96% (*I*^*2*^_Level 2_ = 30%, *I*^*2*^_Level 3_ = 66%); highest *I*^*2*^ = 98% (*I*^*2*^_Level 2_ *=* 30%, *I*^*2*^_Level 3_ = 68%). These sensitivity analyses demonstrate that these results are not driven by one effect-size or one study.

### Moderator analysis

To investigate the potential causes of heterogeneity between studies, we examined several study characteristics as potential moderators (Table 1). Independent-samples *t*-tests were conducted to compare effect-sizes between conditions for each moderator. The effect was larger for memory than comprehension (*G*_diff_ = .24), and when tests were administered verbally rather than in a written format (*G*_diff_ = .43). However, both moderators fell just above the traditional threshold for statistical significance (Table 3). Non-adults also exhibited a larger benefit from narrative texts compared to adults (*G*_diff_ = .23), but this difference was also not statistically significant. Little difference was observed for listening relative to reading (*G*_diff_ = .07), when researchers reported an attempt to control the difficulty (*G*_diff_ = .03) or content across the genres (*G*_diff_ = .02), or for the timing of the test (immediately or after a delay; *G*_diff_ = .03). Note that some of these differences are non-trivial in magnitude, and therefore failure to attain statistical significance may be a function of small sample sizes and/or large amounts of variability. In addition, the *Q* statistic was calculated for each moderator, which represents the residual heterogeneity in effect-sizes when the moderator was taken into account. In all cases, heterogeneity remained after considering the moderator (Table 3).

Because most of these studies measure either memory or comprehension, this introduces a potential confound into our moderator analysis for type of test. To control for this, we conducted a follow-up analysis re-estimating the model using only those six studies that measure both memory and comprehension, effectively controlling for other differences between studies that examine only one or the other. However, results remained the same with the difference between memory and comprehension failing to attain statistical significance (*p* = .31).

### Publication bias

**Fig. 4:**
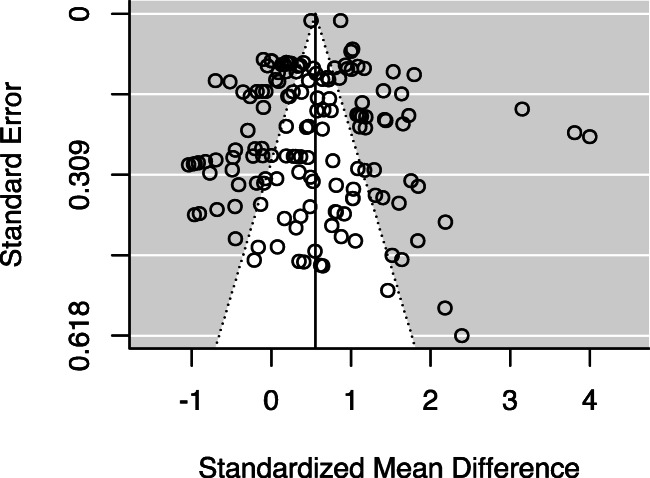
Funnel plot

To diagnose potential publication bias, we produced a funnel plot (Fig. 4), with the lighter region indicating the pseudo 95% confidence limits (± 1.96 × *SE*). When publication bias and heterogeneity are absent, 95% of the effect-sizes should fall within this region and be distributed roughly symmetrically on either side of the average estimate. If publication bias is present, we would expect to see an asymmetry, whereby low precision studies contribute to larger effects, producing more points in the bottom right quadrant, with few matching points in the bottom left quadrant (small effects for low precision studies). In our funnel plot, there is some small evidence of publication bias, with 3 points found in the bottom-right quadrant, but no accompanying points in the bottom-left. In addition, the three largest effect-sizes (values greater than 3, for the standardized mean difference in favour of narrative) are not accompanied by points of equivalent magnitude in the other direction (in favour of expository texts) at the same level of precision.

To further investigate the possibility of publication bias, we conducted an Egger’s regression test, to examine if the standard error of the effect-sizes acts as a moderator of the effect-sizes. Indeed, higher standard errors did predict larger effect-sizes, *b* = 2.68 (95% CI: .57, 4.80), *SE* = 1.04, *p* = .01. In other words, studies with lower precision do tend to find larger effects; in the absence of publication bias, standard error should not be related to the size of an effect. This result is therefore evidence of publication bias within our sample of studies, with studies reporting an advantage for narrative over expository texts when it comes to memory and comprehension perhaps being more likely to be published than null results or the inverse.

## Discussion

Our meta-analysis of 150 effect-sizes (from over 75 unique samples and more than 33,000 participants) found that people had an easier time comprehending and recalling narrative texts compared to expository ones. The average magnitude of this effect was more than a half a standard deviation, with a 95% CI ranging from just more than one-quarter to slightly more than three-quarters of a standard deviation. Moreover, this result appears to be robust, and not driven by any one particular effect-size or study. There was a great deal of variability in these effects, however, almost all of which represents true heterogeneity and not random sampling error. This variability originated primarily from differences between studies. Despite this fact, none of our tests for moderation were statistically significant. This may, however, have been a function of low statistical power. For many of our potential moderators, the difference in effect-sizes for the two groups in question appear to be non-trivial. As an example, the advantage afforded by narrative texts to memory (compared to comprehension) was equivalent to almost one-quarter of a standard deviation (*g* = .24). It is likely a combination of both low sample sizes and large amounts of variability that result in these differences being statistically nonsignificant. The largest difference observed for a moderator was the advantage for verbal testing compared to a written format. This particular finding should be interpreted cautiously, however, as only 21 effect-sizes were based on a verbal test (from six studies), and the CIs around these estimates remain large.

This meta-analysis also provides important guidance for interpreting past studies and guiding future research. For example, there appears to be little evidence that controlling for the difficulty or content across texts has an impact on the effect-size for comprehension and memory. Past work that failed to enact these controls, therefore, may perhaps be viewed in a kinder light based on our results. That said, it cannot be ignored that only a minority of our effect-sizes came from studies in which content was controlled (28%), and more studies with this type of control would be appreciated. In addition, there are clearly topics that are currently under-researched. We located only four studies that examined comprehension or memory after a delay, and only six studies that employed a verbal test of memory or understanding. Both of these areas would benefit from greater attention. Lastly, only eight studies had participants listen to audio versions of the texts, and those that did tended to find a strong advantage for the narrative format. This might also be a good direction for future research.

In general, our confidence in these results is heightened by their convergence with a recent meta-analysis of genre differences for inferential comprehension by Virginia Clinton and her colleagues (Clinton et al., 2020). Their estimated advantage in inferencing for narrative texts is rather similar in magnitude (*G* = .36; 95% CI: .07, .66; based on 38 effect-sizes) to what we observed for our studies of comprehension, more broadly defined (*G* = .48; 95% CI: .21, .75). Similar to our own results, these researchers also did not find evidence of moderation based on age or whether the texts were matched in difficulty. This concordance between the comprehension aspect of our meta-analysis and their work on textual inferences is highly encouraging, especially as it emerged despite different sampling criteria, different meta-analytic methods, and a complete independence of efforts.

Our meta-analysis also uncovered some evidence of publication bias, with asymmetry observed in our funnel plot and the precision of effect-sizes positively predicting effect-size magnitude. That said, gathering and interpreting evidence of publication bias is a difficult undertaking, even more so when there is substantial between-study variability in effect-sizes (Lau, Ioannidis, Terrin, Schmid, & Olkin, 2006). Heterogeneity among effect-sizes can contribute to a statistically significant Egger’s regression test, and an asymmetrical funnel plot, and so these methods may not be appropriate under the conditions observed for our meta-analysis (Terrin, Schmid, Lau, & Olkin, 2003). To explore this evidence for publication bias a bit further, we repeated the Egger’s test on the two sub-samples most likely to contribute to this heterogeneity, separating effect-sizes pertaining to memory from those for comprehension. Based on this test, there was no evidence for publication bias for tests of comprehension (*b* = 1.62; 95% CI: -1.56, 4.81; *SE* = 1.55, *p* = .30), but the same could not be said for memory, *b* = 2.91; 95% CI: .53, 5.29; *SE* = 1.09, *p* = .02. It thus appears that evidence for publication bias originates primarily from investigations of memory. This is also consistent with the prior meta-analysis for inferential comprehension, which found no evidence of publication bias (Clinton et al., 2020). Whether stories are better recalled than essays would therefore appear to warrant further investigation. If additional unpublished research on this topic emerges, it can easily be added to our public data and these analyses re-run based on our posted code.

One limitation of our meta-analysis is that we took a quite broad and inclusive approach. Both comprehension and memory were broadly defined, and were combined in our primary analysis. To be clear, we acknowledge that memory and comprehension are two distinct and unique processes, although they are related. These were combined in our central analysis as they are closely associated in this context, and the theoretical predictions for both were the same. Moreover, we did not find evidence for moderation based on whether memory or comprehension was tested, although the advantage for narrative was stronger for memory. That said, it would not be at all surprising if some readers disagreed with our decision to combine these studies of memory and comprehension. However, we provide estimates for memory and comprehension separately, in reporting our moderation analyses (Table 3). In addition, a real strength of our meta-analysis is that all our data and analysis script are publicly available for download. This means that researchers who disagree with any of our inclusions, categorizations, and groupings can easily make their own decisions and re-run the analysis. Similarly, adding new studies and re-estimating the average effect-size will be a simple process in the future.

In closing, the totality of the evidence available finds that people have an easier time comprehending and recalling information presented in a story compared to that presented in an essay. This has potential implications for a number of disciplines, not least of which is the realm of education. Because texts are an important way in which we encounter new information (Stanovich & Cunningham, 1993), successfully comprehending and retaining this information to build our knowledge of the world is immensely important. To that end, the advantage afforded to narratives over exposition in this domain should be considered whenever possible. We must emphasize, however, that these results should not be interpreted as a suggestion to force all information into a narrative form for pedagogical purposes, especially when such information is not typically presented in this way. Future research is needed to identify the boundary conditions of this narrative advantage, as well as to identify which aspects of a narrative presentation are most important (e.g., prior knowledge, coherence, text schemas, familiarity). It is quite possible that mixed genres like narrative journalism, for example, could hold the key for leveraging the advantages of narrative–its ability to capture interest and communicate experience through imagination – to meet the goals of exposition to inform and educate (van Krieken & Sanders, in press).
